# Opto-APC: Engineering of cells that display phytochrome B on their surface for optogenetic studies of cell-cell interactions

**DOI:** 10.3389/fmolb.2023.1143274

**Published:** 2023-02-20

**Authors:** Marissa Russ, Anna K. Ehret, Maximilian Hörner, Daniel Peschkov, Rebecca Bohnert, Vincent Idstein, Susana Minguet, Wilfried Weber, Björn F. Lillemeier, O. Sascha Yousefi, Wolfgang W. Schamel

**Affiliations:** ^1^ Signalling Research Centres BIOSS and CIBSS, Faculty of Biology, University of Freiburg, Freiburg, Germany; ^2^ Centre for Chronic Immunodeficiency (CCI), Medical Centre Freiburg, Faculty of Medicine, University of Freiburg, Freiburg, Germany; ^3^ Spemann Graduate School of Biology and Medicine (SGBM), University of Freiburg, Freiburg, Germany

**Keywords:** T cell receptor, ligand, receptor, optogenetics, interaction, phytochrome B, SpyCatcher

## Abstract

The kinetics of a ligand-receptor interaction determine the responses of the receptor-expressing cell. One approach to experimentally and reversibly change this kinetics on demand is optogenetics. We have previously developed a system in which the interaction of a modified receptor with an engineered ligand can be controlled by light. In this system the ligand is a soluble Phytochrome B (PhyB) tetramer and the receptor is fused to a mutated PhyB-interacting factor (PIF^S^). However, often the natural ligand is not soluble, but expressed as a membrane protein on another cell. This allows ligand-receptor interactions in two dimensions. Here, we developed a strategy to generate cells that display PhyB as a membrane-bound protein by expressing the SpyCatcher fused to a transmembrane domain in HEK-293T cells and covalently coupling purified PhyB-SpyTag to these cells. As proof-of-principle, we use Jurkat T cells that express a GFP-PIF^S^-T cell receptor and show that these cells can be stimulated by the PhyB-coupled HEK-293T cells in a light dependent manner. Thus, we call the PhyB-coupled cells opto-antigen presenting cells (opto-APCs). Our work expands the toolbox of optogenetic technologies, allowing two-dimensional ligand-receptor interactions to be controlled by light.

## Introduction

The proper reaction of cells to external stimuli is pivotal for life. This is often accomplished by the binding of ligands to receptors, which determines the behavior and fate of the cells. Manipulation of the ligand concentration, the affinity and the half-life of the ligand-interaction, the timing of when the ligand binds or not and the location where the ligand binds, is important to interrogate how cells react to a ligand under changing conditions. One novel technology to temporally modulate the quantity, kinetics and localization of a ligand binding to a receptor, is optogenetics (Fischer et al., 2022; Sánchez and Tampé, 2022).

In non-opsin-based optogenetic systems, light-responsive proteins, such as photoreceptors from plants and cyanobacteria, are the key component. These proteins change their conformation when absorbing light of a certain wavelength and either revert to the original conformation with time or when absorbing light of a different wavelength. In one conformation the photoreceptor binds to a partner protein and in the other conformation it does not. Thus, when the photoreceptor (or a part of it) is fused to protein X and the binding partner (or a part of the binding partner) to protein Y, light illumination can determine when and where these two proteins bind to each other or not (Kolar et al., 2018; Kramer et al., 2021). Thus, in optogenetics these fusion proteins, photoreceptor-X and binding-partner-Y, are expressed in the cell of interest and after light treatment, molecular or cellular effects of the X-Y interaction are measured.

Modulation of the light-intensity can be used to control the amount of X and Y that form the X-Y complex or to control the affinity and half-life of the interaction (Tischer and Weiner, 2019; Yousefi et al., 2019). The timing and localisation of the light illumination can be used to regulate the X-Y interaction with an unprecedentedly high spatio-temporal resolution over several orders of magnitude, ranging from sub-seconds to days and from micrometer to meter (Kolar et al., 2018; Fischer et al., 2022).

Based on pioneering studies (Levskaya et al., 2009; Toettcher et al., 2013), we engineered an optogenetic system in which the interaction of a model ligand with receptors is optogenetically controlled (Baaske et al., 2019; Yousefi et al., 2019). We made use of the plant photoreceptor phytochrome B (PhyB) of *Arabidopsis thaliana* that binds to the PhyB-interacting factor (PIF) when illuminated with red light (Rockwell et al., 2006; Bae and Choi, 2008; Smith et al., 2016). With far-red light the PhyB-PIF interaction is broken. We used the first 651 amino acids of PhyB (PhyB_1-651_). This N-terminal part includes the cGMP phosphodiesterase/adenyl cyclase/FhlA (GAF) region which binds with its conserved cysteine to a light-responsive chromophore. Upon photon absorption, the chromophore molecule undergoes a photochemical reaction, causing a rearrangement of the protein’s structure, resulting in changes in the interaction of PhyB with PIF (Rockwell et al., 2006; Burgie and Vierstra, 2014; von Horsten et al., 2016). Additionally, we used the first 100 amino acids of PIF6, which we mutated to be transported by the secretory pathway to the cell surface (PIF^S^).

In our system, PhyB_1-651_ tetramers served as a ligand for a T cell receptor (TCR) (Yousefi et al., 2019), which itself was fused to GFP and PIF^S^ (GFP-PIF^S^-TCR). In this system, the PhyB_1-651_ ligand binds with red light and detaches with far-red light. Furthermore, the intensity of red light determines the cycling rate of PhyB between the PIF binding and non-binding states (Mancinelli, 1994; Smith et al., 2016) and thus the half-life of the ligand-TCR interaction. We showed that the binding half-life alone can determine whether a TCR ligand acts as an agonist leading to T cell activation (long half-life) or acts as an antagonist (short half-life) (Yousefi et al., 2019; Yousefi et al., 2021).

Under physiological conditions the ligand for the TCR, peptide-MHC, is a transmembrane protein displayed on the surface of the antigen presenting (or target) cell (Schamel et al., 2019). Thus, our current three-dimensional soluble ligand system does not recapitulate the complexity of a two-dimensional receptor-ligand interaction when the ligand-bearing and the receptor-expressing cells contact to each other.

**FIGURE 1:**
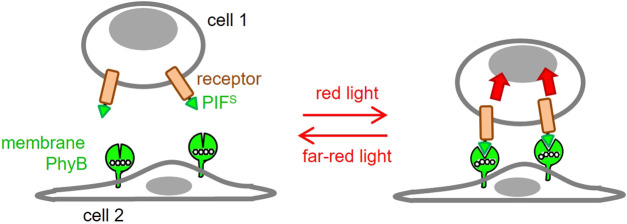
Use of optogenetics to engineer 2D ligand-receptor pairs. Cells displaying a phytochrome B fragment (PhyB_1-651_) on their membrane resemble settings in which the ligand is cell bound. This ligand will bind to a receptor fused to PIF^S^ at red light but not at far-red light conditions. This will resemble the 2D interactions seen in many ligand-receptor pairs.

In this work, we aimed to develop an optogenetic system, in which the ligand (PhyB_1-651_) is displayed on the surface of a cell (Figure 1), rather than being in solution. Our goal was to engineer an antigen presenting cell (APC) for optogenetic control of two-dimensional ligand-receptor interactions.

## Materials and methods

### Molecular cloning

All plasmids generated in this study were created using standard molecular cloning techniques, such as polymerase chain reaction, restriction enzyme digestion and ligation or Gibson assembly (Gibson et al., 2009). Cloning strategies, plasmids and nucleotide sequences are given in the supplementary information. The cloning of all plasmids was verified by restriction enzyme digestion and Sanger sequencing.

### Protein expression and purification

PhyB-mCherry-SpyTag and PhyB-SpyTag contain the first 651 amino acids of PhyB from *Arabidopsis thaliana* as we and others have used it before (Levskaya et al., 2009; Toettcher et al., 2013; Yousefi et al., 2019), fused to mCherry (or not), the third generation of the SpyTag (Keeble et al., 2019) and a His_6_ tag. PhyB-SpyTag was produced in *E. coli* flask cultures as described (Hörner et al., 2020). PhyB-mCherry-SpyTag was produced by high-cell-density *E. coli* fermentation as described (Hörner et al., 2019). Both proteins were purified by Ni-NTA chromatography followed by a buffer exchange to PBS supplemented with 0.5 mM TCEP as described (Hörner et al., 2020). GFP-PIF contains moxGFP (Costantini et al., 2015) fused to the first 100 amino acids of PIF6 from *Arabidopsis thaliana* with the C9S and C10S mutations followed by a His_6_ tag and was purified by standard conditions.

### Spectra

The light-dependent absorbance spectra of PhyB-mCherry-SpyTag and PhyB-SpyTag were recorded as described (Hörner et al., 2020).

### Cell lines and lentiviral transduction

Human embryonic kidney (HEK-293T) cells and the fibroblast cell line DAP-DR1-ICAM1, stably transfected with HLA class II DR1 and ICAM-1 molecules (provided by M. Owen), and their transductants, were grown in RPMI 1640 medium supplemented with 10% fetal bovine serum, 10 mM HEPES, 100 U/ml penicillin and 100 μg/mL streptomycin (all Thermo Fisher) at 37°C and 5% CO_2_ (Schamel et al., 2005). The human B cell line Raji and the human T cell line Jurkat E6.1 expressing the GFP-PIF^S^-TCR (Yousefi et al., 2019) were cultivated in the same conditions.

Lentiviral transduction was done as described earlier (Hartl et al., 2020). Briefly, HEK-293T cells were transfected with the lentiviral packaging plasmid pCMV dR8.74, the envelope plasmid pMD2 VSVG (both gifts from Didier Trono) and the plasmid encoding for the protein of interest by calcium phosphate precipitation. Lentiviral particles were produced for 48 h, harvested, filtered through a 0.45 µm syringe filter and concentrated by overnight centrifugation at 3,000 *g* at 4°C through a 20% (w/v in PBS) sucrose cushion. The viral particles were resuspended in medium using 1/100th of the harvested volume. Cells were transduced with different dilutions of concentrated lentiviral particles and 48 h after transduction, surface expression and cell viability were analyzed by flow cytometry.

### Protein alkylation with iodoacetamide

The PhyB-mCherry-SpyTag protein was diluted with 0.1 M Tris-HCl, 150 mM NaCl, pH 8.0 and concentrated by centrifugation at 4,000 *g* in a spin-column-concentrator (10 kDa) three times to replace the PBS with Tris-HCl. Then 10 mM TCEP was added and incubated for 30 min at room temperature to reduce the cysteines. Next, the cysteines were alkylated with 100x molar excess of iodoacetamide (IAA) over PhyB and incubated for 1 h at room temperature in the dark. The IAA was removed and the buffer changed to PBS by dilution and concentration in a spin-column-concentrator (10 kDa). 10 mM DTT was added and incubated for 1 h before removing the DTT with PBS by centrifugation with a spin column. The alkylated protein was stored at 4°C.

### Coupling of PhyB-mCherry-SpyTag to cells expressing SpyCatcher-TMD-BFP

The medium of HEK-293T or DAP-DR1-ICAM1 cells expressing SpyCatcher-TMD-BFP was removed and either 10 μg/mL PhyB-SpyTag or 10 μg/mL PhyB-mCherry-SpyTag were added in 50 µL medium with 0.4 mM TCEP. After incubation for 30 min the protein suspension was removed and new medium was added.

### Illumination devices

Two different illumination devices were used to illuminate the cells with different wavelengths. Both devices use light-emitting diodes (LEDs). The first device is a LED box, with a panel of far-red and red LEDs in its lid (Yousefi et al., 2019). The box is ventilated and placed in a 37°C, CO_2_ incubator with cooling with a function. The second illumination device was the optoPlate-96 illuminator build according to Bugaj and Lim for a 96-well plate (Bugaj and Lim, 2019). The optoPlate-96 allows individual illumination of each well with far-red and red light and was programmed using optoConfig-96 (Thomas et al., 2020). A black-walled cell culture plate on a microwell plate adaptor and a light-isolating lid prevent light contamination of neighboring wells, while the thermal management component removes the heat from the LEDs by distributing it. Both devices are controlled by an Arduino microcontroller.

### Binding of GFP-PIF to cells coupled to PhyB-mCherry-SpyTag

5 × 10^5^ cells coupled to PhyB-mCherry-SpyTag were detached using PBS with 0.1 mM EDTA and transferred to a 96-well U-bottom plate. After washing with 180 µL PBS, 1% FBS and 0.1 mM EDTA, the cells were resuspended in the same buffer including 0.1 mM TCEP and 10 μg/mL GFP-PIF protein per well. All following steps, including the measurement with the flow cytometer, were performed in the dark or under green light. The cells were illuminated with either red or far-red light of 50% light intensity for 1 min (this corresponds to saturating conditions). After 30 min incubation in the dark at 4°C the cells were washed with PBS, 1% FBS, 0.1 mM EDTA and resuspended in 100 µL of the same buffer. The bound GFP-PIF was quantified by flow cytometry.

### Flow cytometry

Cells were stained for surface proteins according to standard protocols. Briefly, cells were washed once with washing buffer (PBS with 1% FBS), then incubated for 30 min at 4°C in a diluted solution of the desired antibodies. Afterwards the cells were washed twice and analyzed on a MACSQuant X or Attune NxT flow cytometer. The antibodies used in this study were the AlexaFluor647-conjugated anti-HA tag antibody (HA.11 from BioLegend), and anti-His6 antibodies (Invitrogen) followed by FITC-coupled anti-mouse IgG (SouthernBiotech) and PE-Cy7-conjugated anti-CD69 (BioLegend). Data were analysed with the FlowJo software.

### Stimulation of GFP-PIF^S^-TCR-expressing jurkat cells

5 × 10^4^ SpyCatcher-TMD-BFP-expressing HEK293T cells per well were seeded in RPMI medium supplemented with 1% FCS in a F-bottom 96 well plate (black-walled clear bottom, Greiner Bio-One) and incubated over night at 37°C, 5% CO_2._ The cells were loaded with 10 μg/mL PhyB-mCherry-SpyTag for 30 min at 37°C. Then the PhyB-containing solution was removed and 5 × 104 Jurakt T cells in RPMI medium with 10% FCS were added to the wells. The plate was illuminated with far-red light before starting the different light programs using the opto-Plate-96 (Bugaj and Lim, 2019). After the stimulation time, cells were harvested, stained and measured by flow cytometry.

### Microscopy

4 × 10^5^ of detached, soluble or 7.5 × 10^4^ adherend HEK293T cells expressing SpyCatcher-TMD-BFP were loaded with PhyB-mCherry-SpyTag as described in the previous paragraph. Cells were transferred onto poly-L-lysine-coated microscopy chambers (LAB-TEK) and 100 nM recombinant GFP-PIF was added. Cells were illuminated with either red or far-red light for 30 s to shift PhyB into the PIF-binding or non-binding conformation, respectively. Samples were incubated for 20 min and imaging media was removed. Cells were fixed for 20 min in 4% PFA at room temperature. After fixation, PFA solution was replaced with 200 µL PBS and samples were imaged by confocal microscopy (Zeiss LSM 880).

## Statistics

In this study, all graphs represent three or more replicates. The uncertainties of these experiments are shown by the standard error of the mean (SEM). A Student’s t-test or one-way ANOVA was applied to calculate the statistical significance of a difference when comparing two conditions; ns: *p* > 0.05; *: *p* < 0.05; **: *p* < 0.01; ***: *p* < 0.001; ****: *p* < 0.0001.

## Results

### PhyB cannot be expressed as a transmembrane protein on the cell surface

In this work, we aimed to design a PhyB protein that is bound to a mammalian cell surface, in order to stimulate PIF-bearing receptors on an opposing cell (Figure 1). As a first attempt, we cloned PhyB_1-651_ as a chimeric transmembrane protein using the transmembrane region of human MHC class I and a cytosolic blue fluorescence protein (BFP, Supplementary Figure S1A). However, this protein was not transported to the cell surface in human embryonic kidney (HEK-293T) cells as assayed by flow cytometry and microscopy (not shown). PhyB is produced in the cytoplasm in plant cells, where N-linked glycosylations and disulfide bond formation do not occur. However, our PhyB_1-651_ folds in the lumen of the endoplasmic reticulum of mammalian cells, where glycosylations and cysteine oxidations occur, possibly hindering the folding of PhyB_1-651_ and its consequent expression on the cell surface. Thus, we generated a number of PhyB_1-651_ variants where the glycosylation site and various cysteines were mutated. However, in none of these cases, PhyB_1-651_ was expressed on the cell surface (Supplementary Figure S1 and not shown). Hence, we aimed to look for an alternative strategy.

### Coupling of PhyB to the surface of cells using the SpyTag-SpyCatcher system

Since PhyB_1-651_ conjugated to the chromophore phycocyanobilin can be expressed with high yield in *E. coli* (Hörner et al., 2019), we intented to couple bacterially expressed PhyB_1-651_ to cells. The third generation of the SpyTag-SpyCatcher system was chosen (SpyTag003-SpyCatcher003 (Keeble et al., 2019)), because it can be used to irreversibly conjugate two proteins by forming an amide bond between the SpyTag and SpyCatcher proteins and the reaction occurs under different pH and temperature conditions (Zakeri et al., 2012).

**FIGURE 2:**
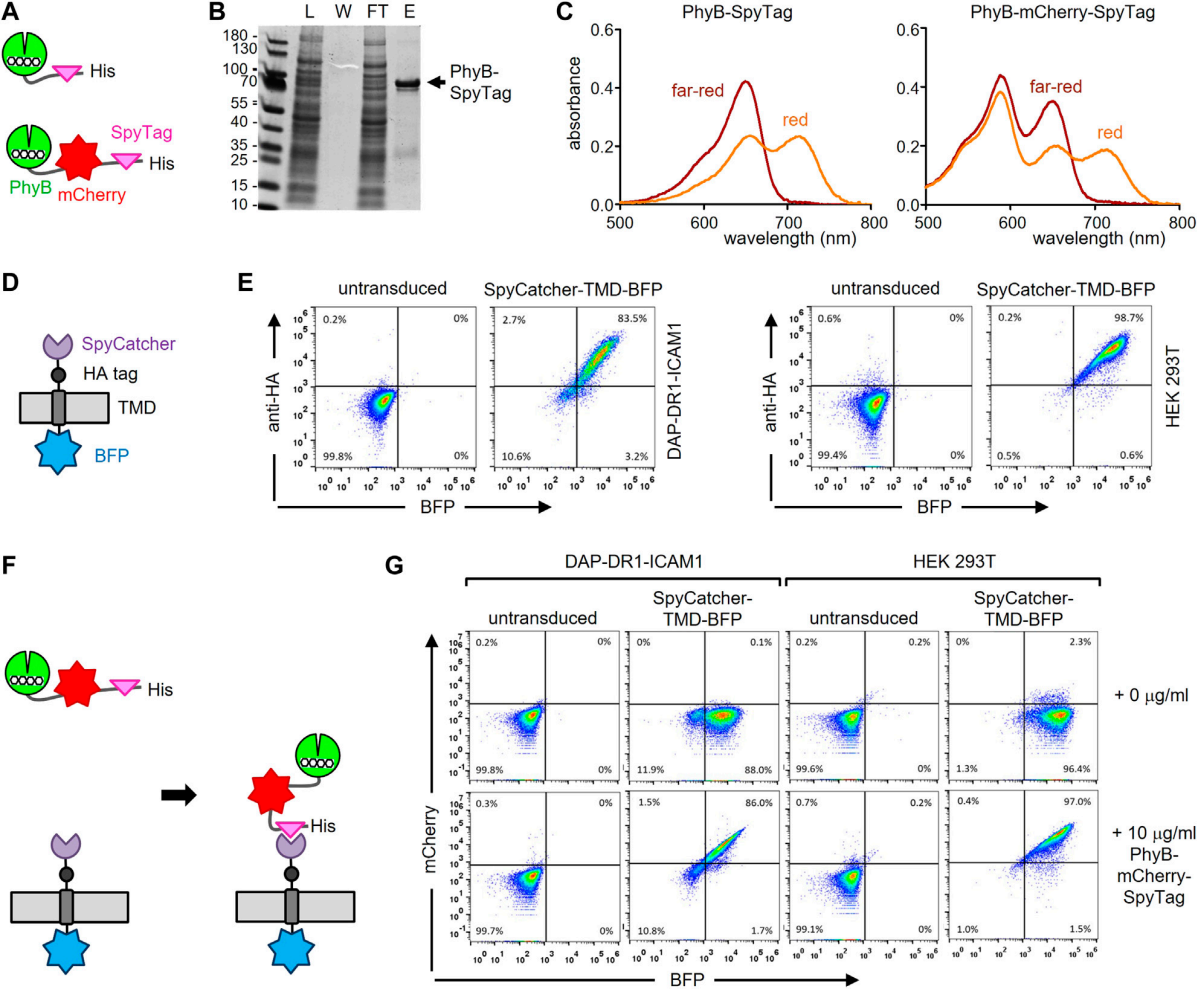
Engineering the PhyB-SpyTag and SpyCatcher-TMD-BFP system. **(A)** Scheme of the PhyB-SpyTag constructs without and with mCherry. **(B)** PhyB-SpyTag was expressed in bacteria and purified. The lysate (L), washing step (W), flow through (FT) and eluate **(E)** fractions of the Ni^2+^-affinity chromatography were separated by SDS-PAGE and proteins visualized by Coomassie (n = 2). **(C)** Purified PhyB-SpyTag and PhyB-mCherry-SpyTag were illuminated with 660 nm (red) or 740 nm (far-red) light and the absorbance spectra measured. **(D)** Scheme of the SpyCatcher-TMD-BFP construct. **(E)** SpyCatcher-TMD-BFP was lentivirally expressed in DAP-DR1-ICAM1 and HEK-293T cells, stained with an AlexaFluor647-coupled anti-HA tag antibody, and measured by flow cytometry. Dot plots of the anti-HA stain and BFP fluorescence intensity from untransduced and transduced cells are shown (n > 3). **(F)** Illustration of the covalent coupling of PhyB-mCherry-SpyTag to SpyCatcher-TMD-BFP expressed on the cell. **(G)** DAP-DR1-ICAM1 and HEK-293T cells expressing SpyCatcher-TMD-BFP or not (untransduced) were incubated with 10 μg/mL of the PhyB-mCherry-SpyTag protein or not (0 μg/mL) for 20 min at 37°C in RPMI 1640 medium. After washing, the cells were measured by flow cytometry and the fluorescence intensities of mCherry and BFP are shown (n > 3).

Firstly, a fusion protein consisting of PhyB_1-651_, the SpyTag003 (in the remainder of the work referred to as SpyTag) and a His_6_-tag for purification of the protein was designed; in a second version the fluorescent protein mCherry was also included (Figure 2A and Supplementary Figure S2A). Because the proteins were produced in *E. coli*, the expression plasmids also contained the enzymes heme oxygenase and PCB:ferredoxin oxidoreductase, which are needed for the synthesis of phycocyanobilin (PCB), which serves as the light-responsive chromophore of PhyB. Consequently, the PhyB-SpyTag and PhyB-mCherry-SpyTag proteins were produced and purified from the bacterial lysates using Ni2+-affinity chromatography. The lysate, the wash, flow-through and eluate fractions were analyzed by SDS-PAGE and Coomassie staining. PhyB-SpyTag possessed the expected size of 74 kDa and PhyB-mCherry-SpyTag of 100 kDa (Figure 2B and not shown). In both cases impurities were also detected. The purified PhyB_1-651_ was able to switch between its two conformations, since examining the photoisomerization by recording the absorbance spectra after illumination with red or far-red light resulted in the expected curves (Figure 2C) (Mukougawa et al., 2006). The additional peak at 585 nm of PhyB-mCherry-SpyTag was caused by mCherry. In conclusion, both PhyB-SpyTag and PhyB-mCherry-SpyTag were recombinantly produced and purified, and switched their conformation with light, indicating proper folding and functionality.

Secondly, we engineered a chimeric protein being expressed on the surface of a cell and encompassing the SpyCatcher003 (here referred to as SpyCatcher) extracellularly. This protein contained the signal peptide of human MHC class I, the SpyCatcher, an HA tag, the transmembrane domain (TMD) of human MHC class I and BFP at the C-terminus (Figure 2D; Supplementary Figure S2B). The fibroblast cell line DAP-DR1-ICAM1 (Schamel et al., 2005) and the HEK-293T cell line were lentivirally transduced to express the SpyCatcher-TMD-BFP construct. Cells were stained with an anti-HA tag antibody and measured by flow cytometry (Figure 2E). Untransduced cells served as a negative control. In both cell lines, the construct was expressed (as detected by the BFP fluorescence) and transported to the cell surface (as detected by the anti-HA stain). In the HEK-293T cells, the expression level was higher than in the DAP-DR1-ICAM1 cells (as seen by the mean fluorescence intensities (MFI) of BFP and of the anti-HA stain, Supplementary Figure S2C). The expression level in the HEK-293T cells was also higher than the one in the human Raji B cell line (not shown).

Thirdly, we tested whether we can couple PhyB-mCherry-SpyTag to the SpyCatcher-TMD-BFP-expressing cells (Figure 2F). To this end, DAP-DR1-ICAM1 cells expressing SpyCatcher-TMD-BFP were incubated without or with 10 μg/mL of the PhyB-mCherry-SpyTag protein for 20 min. The cells were then washed and analyzed by flow cytometry for BFP and mCherry fluorescence (Supplementary Figure S2G, left panels). As a control, untransduced cells were used. SpyCatcher-TMD-BFP-expressing cells that were incubated with the PhyB-mCherry-SpyTag protein showed an increase in the mCherry fluorescence compared to the other conditions, indicating that PhyB-mCherry-SpyTag was coupled to the cells. There was a correlation between the BFP and mCherry fluorescence intensities, suggesting that the cells with more SpyCatcher-TMD-BFP could bind more PhyB-mCherry-SpyTag. A similar result was obtained using the HEK-293T cells (Figure 2G, right panels), with the difference that more PhyB-mCherry-SpyTag coupled to the cells than when using the DAP-DR1-ICAM1 cells. Hence, for the further experiments the HEK-293T SpyCatcher-TMD-BFP transduced cells were used.

To optimize the coupling process, we varied the coupling time and the PhyB-mCherry-SpyTag concentration using HEK-293T SpyCatcher-TMD-BFP cells (Supplementary Figure S2D, E). The amount of coupled PhyB-mCherry-SpyTag increased with higher concentrations and longer coupling times. For 10 and 32 μg/mL PhyB-mCherry-SpyTag a saturation of the coupling was reached upon 120 min. Approximately 70% coupling was seen with 10 μg/mL after 30 min. Thus, for further experiments this condition was chosen.

### Binding of a PIF-fused protein to the PhyB-coupled cells

**FIGURE 3:**
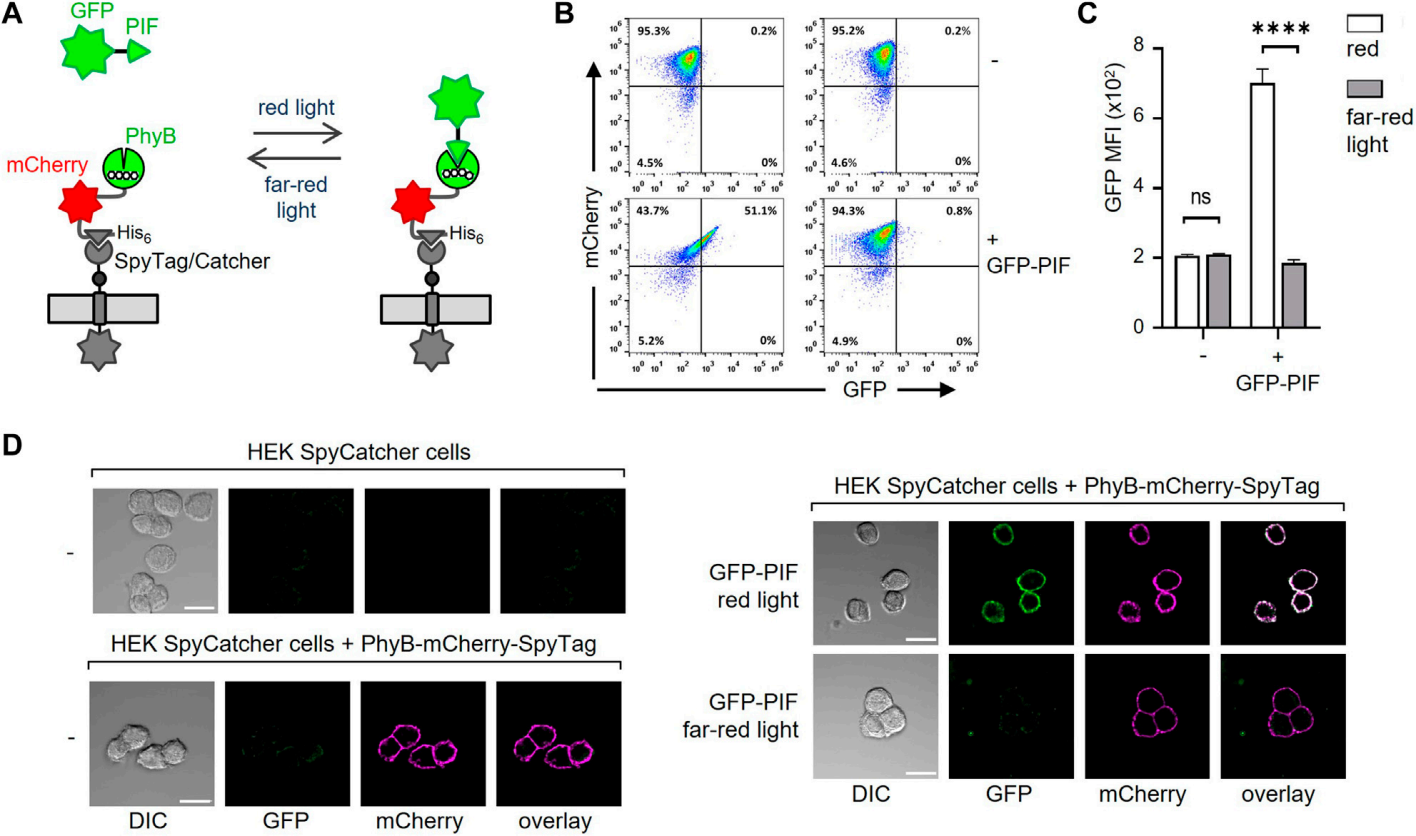
Binding of GFP-PIF to PhyB-mCherry-SpyTag-coupled cells. **(A)** Illustration of the binding of GFP-PIF to PhyB-mCherry-SpyTag, which was coupled to SpyCatcher-TMD-BFP-expressing cells. **(B)** SpyCatcher-TMD-BFP-expressing HEK-293T cells were loaded with 10 μg/mL PhyB-mCherry-SpyTag for 30 min at 37°C and 5% CO_2_. After washing, the cells were incubated with 10 μg/mL GFP-PIF in PBS, 1% FBS, 0.1 mM EDTA and 0.1 mM TCEP. The samples were illuminated for 1 min with either 660 nm (red) or 740 nm (far-red) light and left in the dark for 30 min. The cells were then washed and analyzed by flow cytometry in the dark (n > 3). **(C)** Quantification of the GFP MFI from **(B)**. **(D)** SpyCatcher-TMD-BFP-expressing HEK-293T cells were detached with EDTA in PBS and loaded or not with PhyB-mCherry-SpyTag as indicated. 100 nM GFP-PIF was added (right panels) or not (left panels) and cells were illuminated with red, 660 nm or far-red, 740 nm light and incubated for 20 min in the dark. Cells were imaged by confocal microscopy. Scale bar represents 20 µm (n > 3).

After coupling of PhyB-mCherry-SpyTag to SpyCatcher-TMD-BFP on the cells, the functionality of PhyB was tested by light-controlled PIF binding. To this end, a moxGFP-PIF61-100 (C9S, C10S)-His6 fusion protein (referred to as GFP-PIF) was used to validate binding to the HEK-293T cells displaying PhyB_1-651_ under red light (Figure 3A). No binding of GFP-PIF should occur under far-red light. The PhyB_1-651_-displaying HEK-293T cells were incubated with 10 μg/mL GFP-PIF in PBS. The samples were illuminated at saturating conditions for 1 min with either red or far-red light to switch PhyB_1-651_ to the PIF-binding or PIF-not-binding states, respectively. Then the samples were left in the dark for 30 min to allow GFP-PIF to bind or not. The cells were washed and analyzed by flow cytometry in the dark to avoid conversion of PhyB_1-651_ and a possible loss of the GFP-PIF binding (Supplementary Figure S3B, C). The mCherry fluorescence shows that most cells were coupled to PhyB-mCherry-SpyTag; and those cells gained GFP fluorescence when red light was used, compared to far-red. This shows that PIF-GFP could bind to the PhyB_1-651_-coupled cells in a light-dependent manner.

We also tested the binding of PhyB-mCherry-SpyTag to the HEK-293T SpyCatcher-TMD-BFP cells by microscopy and found that it nicely localized to the cell surface. Furthermore, in a light-dependent manner GFP-PIF^S^ bound to the cells, but only when PhyB-mCherry-SpyTag was present (Figure 3D; Supplementary Figure S3). Therefore, our new system works as we had intended.

### PhyB_1-651_ looses its capacity bind to GFP-PIF after 2 hours in medium at 37°C

**FIGURE 4:**
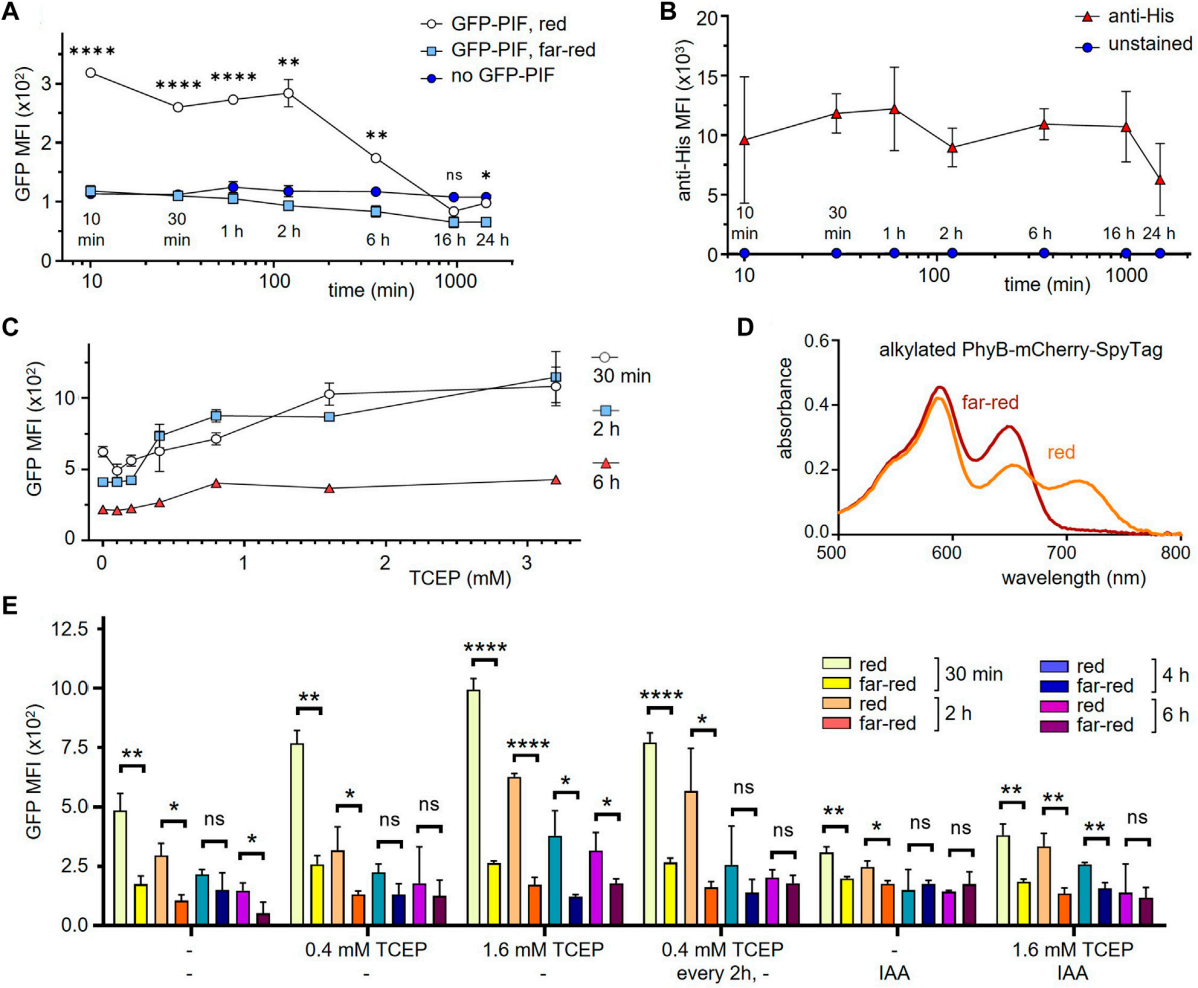
Improving the functionality of PhyB_1-651_ coupled to cells in mammalian cell culture conditions. **(A)** HEK-293T SpyCatcher-TMD-BFP cells were coupled to 10 μg/mL PhyB-mCherry-SpyTag for 30 min and then incubated in RPMI medium with 1% FBS at 37°C for the time points indicated. Subsequently, cells were treated with 10 μg/mL GFP-PIF in PBS and illuminated for 1 min with 660 nm (red) or 740 nm (far-red) light, and left in the dark for 30 min. After washing, the cells were analyzed by flow cytometry and the MFI of GFP is displayed (*n* = 2). **(B)** In parallel, the cells from **(A)** without incubation with GFP-PIF were stained with an anti-His tag antibody as above and the MFI of this stain is shown (*n* = 2). **(C)** The experiment was performed as in **(A)**, but adding the indicated amounts of TCEP during the incubation times. GFP-PIF binding was assessed with 660 nm light illumination (*n* = 2). **(D)** PhyB-mCherry-SpyTag was treated with IAA for 1 h, illuminated with 660 nm (red) or 740 nm (far-red) light and the absorbance spectra was measured. **(E)** The experiment was performed as in **(C)** at the indicated conditions (*n* = 1).

To test how long PhyB_1-651_ stays in a functional form on the cell surface during cell culture, HEK-293T SpyCatcher-TMD-BFP cells coupled to PhyB-mCherry-SpyTag were incubated for different times points ranging from 10 min to 24 h at 37°C in medium. Then cells were incubated with 10 μg/mL GFP-PIF and illuminated for 1 min with red or far-red light, and left in the dark for 30 min. After washing, cells were analyzed by flow cytometry (Figure 4A). Using red light, GFP-PIF was bound when cells were incubated less than 2 h before addition of GFP-PIF. At 6 h incubation the GFP-PIF binding to the cells was reduced and at 16 h below the detection limit. Since PhyB-mCherry-SpyTag was still present (as shown by an anti-His6-tag stain, Figure 4B), this indicated that the majority of PhyB_1-651_ had lost is capability to bind to PIF when present in cell culture for longer than 2 h. As expected, under far-red light, GFP-PIF did not bind under any conditions (Figure 4A).

### Prolonging the functional state of PhyB_1-651_ when coupled to cells

One possibility to explain the loss of function under mammalian cell culture conditions could stem from oxidation of PhyB. To test this, the reducing agend TCEP was added to the medium, in order to slow down the oxidation of PhyB, which might render PhyB inactive. HEK-293T SpyCatcher-TMD-BFP cells coupled to PhyB-mCherry-SpyTag were incubated for 30 min, 2 h, and 6 h in the presence of different TCEP concentrations. Binding of GFP-PIF under red light was quantified as above. The GFP-PIF binding increased with higher TCEP concentrations (Figure 4C), indicating that we can prolong the time during which PhyB is functional. However, even at its highest concentration TCEP could not fully prevent loss of PhyB activity. Thus, we tested further methods to prevent oxidation of cysteines. The alkylating agent iodoacetamide (IAA) is commonly used to prevent oxidation of cysteines by binding to the cysteine thiol group. Therefore, we alkylated PhyB-mCherry-SpyTag using an excess of IAA and tested whether the alkylated form could switch between its two photostates using red or far-red light. The absorbance spectra did not show a difference to the non-alkylated PhyB-mCherry-SpyTag (Figure 4D). Thus, we concluded that IAA treatment preserves the function of PhyB.

Next, we evaluated different treatments of PhyB with IAA combined with TCEP in their capability to preserve the PIF-binding function of PhyB. To this end, SpyCatcher-TMD-BFP-expressing HEK-293T cells were loaded with either alkylated PhyB-mCherry-SpyTag or untreated PhyB-mCherry-SpyTag. In one condition, samples were treated with 0.4 mM TCEP every 2 h. After 30 min, 2 h, 4 h, and 6 h, GFP-PIF in it is active conformation was added to the cells as above and GFP-PIF binding was quantified by flow cytometry (Figure 4E). Each treatment condition shows decreased GFP-PIF binding after longer incubation times, indicating that PhyB functionality is lost with time, even under the alkylating and reducing conditions. In fact, IAA did not improve GFP-PIF binding, but had the opposite effect. The best preservation of PhyB function was seen with 1.6 mM TCEP. However, for longer times this high concentration decreases the viability of the cells (not shown). Thus, we decided to use 1.6 mM TCEP, when PhyB-mCherry-SpyTag was present, during experiments lasting 6 h or less. For longer incubations, we use 0.4 mM TCEP.

### Stimulation of GFP-PIF^S^-TCR T cells by cells coupled to PhyB_1-651_

**FIGURE 5:**
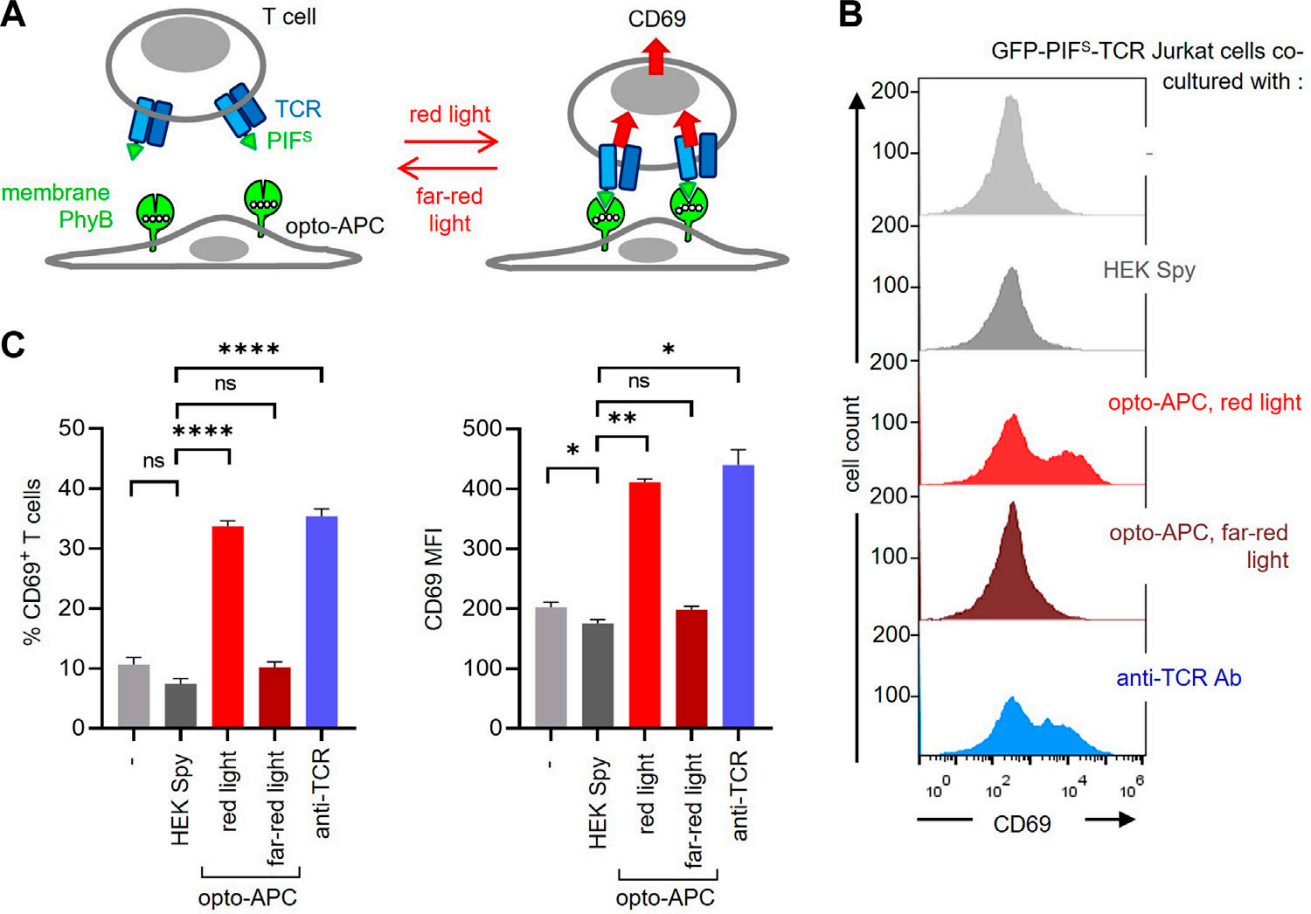
Stimulation of GFP-PIF^S^-TCR T cells by the PhyB_1-651_-coupled HEK-293T cells. **(A)** Scheme of the HEK-293T SpyCatcher-TMD-BFP cells coupled to PhyB-mCherry-SpyTag, called opto-APCs, stimulating GFP-PIF^S^-TCR-expressing T cells at red light (630 nm), but not at far-red light (780 nm). **(B)** GFP-PIF^S^-TCR-expressing Jurkat T cells were co-cultured at 37°C for 6 h with the following cells: no cells (−), un-coupled HEK-293T SpyCatcher-TMD-BFP cells (HEK Spy), opto-APCs with a 30 s 630 nm light illumination every 15 min (opto-APC, red light), opto-APCs with 1 min 780 nm light every 15 min (opto-APC, far-red light), or with the stimulating anti-TCR antibody Jovi3. Cells were stained with a PE-Cy7-conjugated anti-CD69 antibody and measured by flow cytometry. anti-CD69 fluorescence is shown on gated T cells. **(C)** The precent of CD69-positive T cells (left) and the MFI of the anti-CD69 stain (right) are shown for the experiment in **(B)**; n > 3.

Our goal was to use the PhyB_1-651_ of the PhyB-mCherry-SpyTag-coupled cells as a ligand to stimulate a receptor fused to PIF. Hence, the HEK-293T SpyCatcher-TMD-BFP cells coupled to PhyB-mCherry-SpyTag were co-cultured with GFP-PIF^S^-TCR-expressing Jurkat T cells. In this context, the coupled HEK-293T cells serve as antigen-presenting cells (APCs), presenting the ligand (in this case PhyB_1-651_) to the modified TCR (Figure 5A). Hence, from now on we term these modified HEK cells opto-APCs. Stimulation of the GFP-PIF^S^-TCR was quantified by the upregulation of the activation marker CD69 by the T cells (Figure 5A). The 2 cells were co-cultured for 6 h, harvested, stained with a fluorescent anti-CD69 antibody and measured by flow cytometry. After recording, the T cells can be separated from the opto-APCs during the analysis due to their lack of BFP and mCherry fluorescence (Supplementary Figure S4). In the following, we only analysed the T cells. As expected, the GFP-PIF^S^-TCR Jurkat T cells alone (−) or co-cultured with HEK-293T SpyCatcher-TMD-BFP cells not coupled to PhyB_1-651_ (HEK Spy) hardly express CD69 (Fig. 5B and C). When co-cultured with the opto-APCs illuminated with redlight the GFP-PIF^S^-TCR was stimulated as seen by the CD69 expression of the T cells. Under far-red light the T cells did not express CD69 above basal levels. This shows that the ligand-receptor interaction can be controlled by light in our 2D system. As a positive control, stimulation with an anti-TCR antibody also led to CD69 expression by the T cells (Figures 5B,C).

**FIGURE 6:**
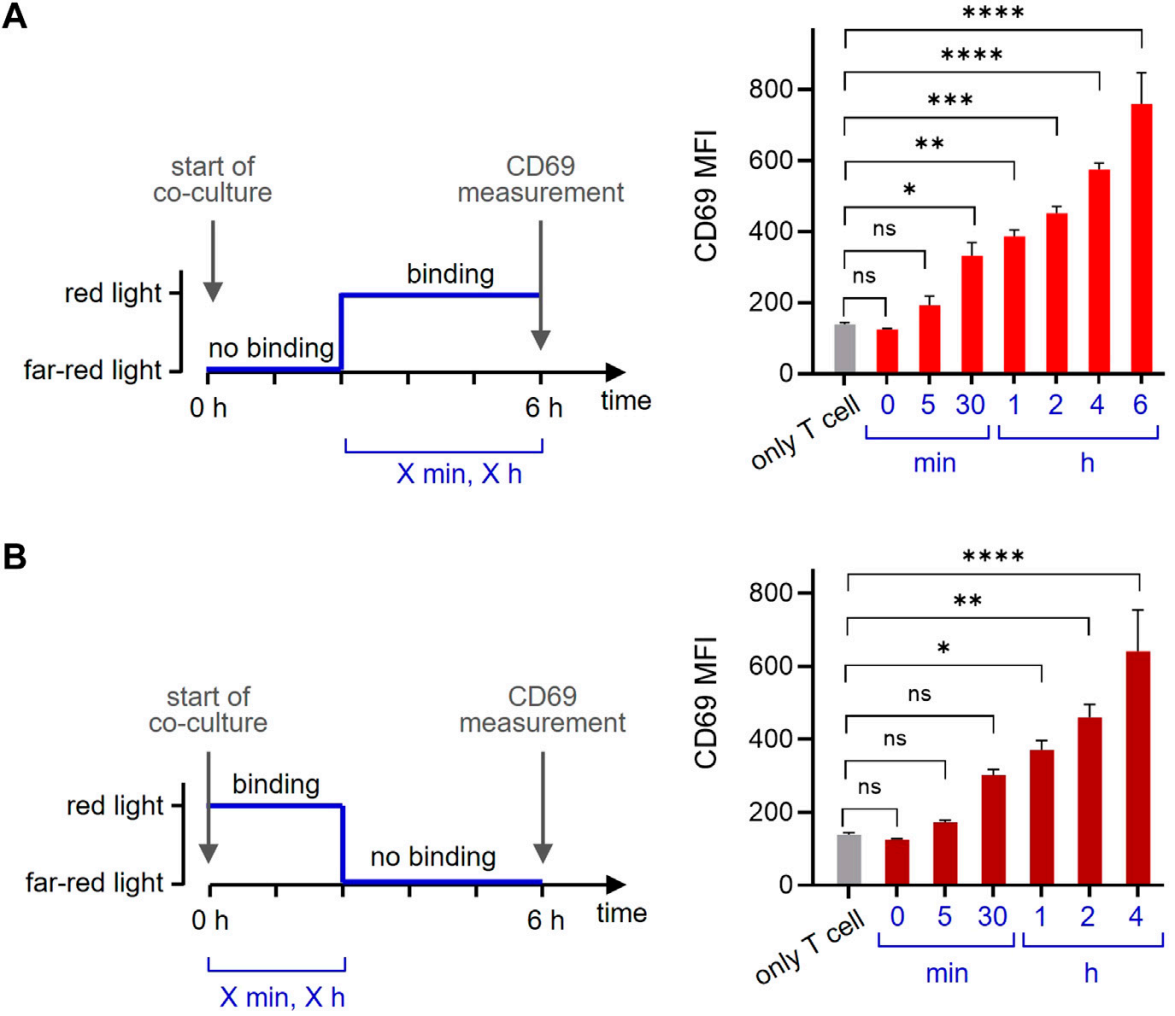
The timing of ligand binding determines the cellular response. **(A)** GFP-PIF^S^-TCR-expressing Jurkat T cells were co-cultured at 37°C for 6 h with no additional cells (only T cells), or with the opto-APCs. At the start of the co-culture, cells were illuminated with 780 nm (far-red) light for 1 min and after 5 h and 55 min (5 min), 5 h and 30 min (30 min), 5h (1 h), 4h (2 h) or 2 h (4 h) cells were exposed to 30 s 630 nm (red) light allowing ligand binding (the ligand binding time is the value given in the brackets and in the right panel). For every light condition pulses were applied every 15 min. The time from the first 630 nm light illumination to the completion of the total 6 h is given. After 6 h, the cells were stained with a PE-Cy7-conjugated anti-CD69 antibody and measured by flow cytometry. After gating on the T cells, the anti-CD69 fluorescence is shown, *n* = 2. **(B)** The experiment was done as in **(A)** with the difference that the co-culture started under 630 nm red light. After 0 min, 5 min, 30 min, 1 h, 2 h or 4 h the cells were exposed to 1 min 780 nm far-red light disrupting ligand binding. After a total of 6 h the cell were measured and analysed.

One of the beauty of the optogenetic system is its reversibility. To make use of this property, we co-cultured the GFP-PIF^S^-TCR T cells with the opto-APCs for 6 h and measured CD69 as before. Firstly, we started with the ligand in the non-binding state using far-red light and then switched to the binding state using red light (Figure 6A, left panel). Letting the ligand bind for 30 min before the measurement slightly increased the amount of CD69 on the cell surface, indicating that the first CD69 molecules can be produced and reach the surface in such a short time (Figure 6A, right panel). Prolonging the time to 1, 2, 4 and 6 h continuously increased the amount of CD69 on the surface. Secondly, we let the ligand bind for a given amount of time (by illumination with red light) and then stopped ligand binding (with far-red light) until the 6 h were complete (Figure 6B, left panel). The data show that 30 min of ligand binding induces CD69 production, so that after another 5.5 h some CD69 molecules can be still detected on the cell surface (Figure 6B, right panel). When binding time was increased and the non-binding time decreased, more CD69 was seen on the surface after 6 h. Together this shows that optogenetics using our newly developed opto-APC can be used to experimentally control the timing of ligand binding to a receptor and then quantifying the downstream response.

## Discussion

Here, we developed an optogenetic system, in which PhyB_1-651_ is coupled to the surface of a mammalian cell, rather than being present in solution. In combination with a second cell that expresses a PIF^S^-receptor construct, this allows to study the impact of two-dimensional ligand-receptor interactions on cellular responses (Figure 1).

Our first attempt in expressing a PhyB_1-651_ as a transmembrane protein was not successful, since all variants that we tested were not transported to the cell surface. Most likely PhyB_1-651_ and its mutants (mutating the N-linked glycosylation site or cysteines) did not pass the quality control system of the endoplasmic reticulum (ER). When PhyB_1-651_ is expressed in the cytosol of mammalian cells, addition of the chromophore PCB to the medium allows formation of a functional PhyB, indicating that PCB crosses the plasma membrane (Levskaya et al., 2009; Toettcher et al., 2013). Thus, PCB might also have entered into the lumen of the ER. However, in contrast to the cytosol, the ER lumen is an oxidizing environment where cysteines either form disulfide bridges or serve as an ER-retention signal (Anelli and Sitia, 2008). Although we mutated most cysteines, we could not mutate C357, since this is the binding site for the chromophore PCB (Wagner et al., 2005). Thus, wrong di-sulfide bridges or an unpaired cysteine might have prevented PhyB_1-651_ from leaving the ER and being transported to the cell surface. Indeed, we have noticed that recombinant PhyB_1-651_ is sensitive to oxidation, in that oxidation destroys the functionality of PhyB.

In our second attempt we used the SpyCatcher-SpyTag pair, since it had been developed to covalently link a fusion protein containing the SpyCatcher to a fusion protein containing the SpyTag (Zakeri et al., 2012). SpyTag and SpyCatcher were obtained by splitting the domain of the *Streptococcus pyogenes* fibronectin-binding protein FbaB that forms a spontaneous isopeptide bond between Lys and Asp. Hence, the SpyTag peptide rapidly forms an amide bond to the SpyCatcher protein once it is bound. Here, we expressed the third generation of the SpyCatcher (Keeble et al., 2019) as a transmembrane protein on the surface of mammalian cells. In parallel, a PhyB_1-651_-SpyTag fusion protein was produced in bacteria and purified according to our previously optimized protocols (Hörner et al., 2019; Hörner et al., 2020). Then PhyB_1-651_-SpyTag, or mostly PhyB_1-651_-mCherry-SpyTag, was coupled to the SpyCatcher-TMD-BFP-expressing cells. Successful coupling was detected by mCherry which then was bound to the SpyCatcher cells. The cell-coupled PhyB_1-651_ was functionally active, since (i) it bound to GFP-PIF under red, but not under far-red light illumination, and (ii) it stimulated GFP-PIF^S^-TCR-expressing T cells with red, but not with far-red light. Hence, our system to establish a cell that displays PhyB_1-651_ on its surface was successful, and in the context of stimulating a TCR we call these cells opto-APCs.

The coupled PhyB was functional on the surface for only a few hours. Firstly, the cells do not express the PhyB protein themselves and thus the turnover of the SpyCatcher-TMD-BFP protein will also limit the amount of PhyB on the surface after coupling. Secondly, we demonstrate here that PhyB’s activity is sensitive to oxidation. PhyB_1-651_ contains 12 cysteines and we speculate that oxidation of one or several of them leads to a loss of PhyB’s function. Thus, we use reducing agents in the production and storage buffer (Hörner et al., 2020; Yousefi et al., 2020). However, when coupled to cells the activity is lost after a few hours. We were able to increase the activity by adding a reducing agent to the cell culture medium, however this is limited by the fact that high concentrations and long treatments of the cells with reducing agents affect the viability of the cells. In our case a concentration of 0.4 mM TCEP seemed to be a good compromise.

We and others have fused binding domains to the ectodomains of several subunits of the TCR (TCRα, TCRβ, CD3γ and CD3ε) either a single chain Fv fragment (Gil et al., 2002; Minguet et al., 2007; Baeuerle et al., 2019), a single domain antibody V region (Baeuerle et al., 2019)or a single strand DNA oligonucleotide (Taylor et al., 2017). Although connected to the TCR with a flexible linker, binding of the corresponding ligands (either as multivalent soluble reagents or on a surface) led to conformational changes at the CD3 subunits (Gil et al., 2002; Minguet et al., 2007; Swamy et al., 2016), subsequent TCR signaling and T cell activation (Minguet et al., 2007; Swamy et al., 2016; Schamel et al., 2017; Taylor et al., 2017; Baeuerle et al., 2019). And thus, our GFP-PIF^S^-TCR is stimulated by soluble PhyB_1-651_ tetramers (Yousefi et al., 2020; Yousefi et al., 2021) and cell-bound PhyB_1-651_. Using our new opto-APCs, we show that the optogenetic approach can be used to experimentally determine the ligand binding time and quantify the downstream cellular response. We find that both the duration of ligand binding and the duration of non-binding impact on the expression of CD69 on the surface of the T cells. As expected, longer binding times and shorter non-binding times favour CD69 appearance on the surface.

In conclusion, with our new system one can optogenetically control ligand-receptor interactions when both proteins are present on a cell. This will allow studying how the dynamics and location of ligand-binding to receptors impact on the intercellular signaling pathways downstream of the receptor and the resulting response of the receptor-expressing cell.

## Data Availability

The original contributions presented in the study are included in the article/Supplementary Material further inquiries can be directed to the corresponding author.

## References

[B1] Anelli T. Sitia R. (2008). Protein quality control in the early secretory pathway. EMBO J. 27, 315–327. 10.1038/sj.emboj.7601974 18216874 PMC2234347

[B2] Baaske J. Mühlhäuser W. W. D. Yousefi O. S. Zanner S. Radziwill G. Hörner M. (2019). Optogenetic control of integrin-matrix interaction. Commun. Biol. 2, 15. 10.1038/s42003-018-0264-7 30652127 PMC6325061

[B3] Bae G. Choi G. (2008). Decoding of light signals by plant phytochromes and their interacting proteins. Annu. Rev. Plant Biol. 59, 281–311. 10.1146/annurev.arplant.59.032607.092859 18257712

[B4] Baeuerle P. A. Ding J. Patel E. Thorausch N. Horton H. Gierut J. (2019). Synthetic TRuC receptors engaging the complete T cell receptor for potent anti-tumor response. Nat. Commun. 10, 2087. 10.1038/s41467-019-10097-0 31064990 PMC6504948

[B5] Bugaj L. J. Lim W. A. (2019). High-throughput multicolor optogenetics in microwell plates. Nat. Protoc. 14, 2205–2228. 10.1038/s41596-019-0178-y 31235951

[B6] Burgie E. S. Vierstra R. D. (2014). Phytochromes: An atomic perspective on photoactivation and signaling. Plant Cell 26, 4568–4583. 10.1105/tpc.114.131623 25480369 PMC4311201

[B7] Costantini L. M. Baloban M. Markwardt M. L. Rizzo M. A. Guo F. Verkhusha V. V. (2015). A palette of fluorescent proteins optimized for diverse cellular environments. Nat. Commun. 6, 7670. 10.1038/ncomms8670 26158227 PMC4499870

[B8] Fischer A. A. M. Kramer M. M. Radziwill G. Weber W. (2022). Shedding light on current trends in molecular optogenetics. Curr. Opin. Chem. Biol. 70, 102196. 10.1016/j.cbpa.2022.102196 35988347

[B9] Gibson D. G. Young L. Chuang R.-Y. Venter J. C. Hutchison C. A. Smith H. O. (2009). Enzymatic assembly of DNA molecules up to several hundred kilobases. Nat. Methods 6, 343–345. 10.1038/nmeth.1318 19363495

[B10] Gil D. Schamel W. W. A. Montoya M. Sánchez-Madrid F. Alarcón B. (2002). Recruitment of Nck by CD3 epsilon reveals a ligand-induced conformational change essential for T cell receptor signaling and synapse formation. Cell 109, 901–912. 10.1016/s0092-8674(02)00799-7 12110186

[B11] Hartl F. A. Beck-Garcìa E. Woessner N. M. Flachsmann L. J. Cárdenas R. M.-H. V. Brandl S. M. (2020). Noncanonical binding of Lck to CD3ε promotes TCR signaling and CAR function. Nat. Immunol. 21, 902–913. 10.1038/s41590-020-0732-3 32690949

[B12] Hörner M. Gerhardt K. Salavei P. Hoess P. Härrer D. Kaiser J. (2019). Production of phytochromes by high-cell-density *E. coli* fermentation. ACS Synth. Biol. 8, 2442–2450. 10.1021/acssynbio.9b00267 31526004

[B13] Hörner M. Yousefi O. S. Schamel W. Weber W. (2020). Production, purification and characterization of recombinant biotinylated phytochrome B for extracellular optogenetics. Bio Protoc. 10, e3541. 10.21769/bioprotoc.3541 PMC7842835 33659515

[B14] Keeble A. H. Turkki P. Stokes S. Khairil Anuar I. N. A. Rahikainen R. Hytönen V. P. (2019). Approaching infinite affinity through engineering of peptide-protein interaction. Proc. Natl. Acad. Sci. U. S. A. 116, 26523–26533. 10.1073/pnas.1909653116 31822621 PMC6936558

[B15] Kolar K. Knobloch C. Stork H. Žnidarič M. Weber W. (2018). OptoBase: A web platform for molecular optogenetics. ACS Synth. Biol. 7, 1825–1828. 10.1021/acssynbio.8b00120 29913065

[B37] Kramer M. M. Lataster L. Weber W. Radziwill G. (2021). Optogenetic Approaches for the Spatiotemporal Control of Signal Transduction Pathways. Int. J. Mol. Sci. 22 (10), 5300. 10.3390/ijms221053000 34069904 PMC8157557

[B16] Levskaya A. Weiner O. D. Lim W. A. Voigt C. A. (2009). Spatiotemporal control of cell signalling using a light-switchable protein interaction. Nature 461, 997–1001. 10.1038/nature08446 19749742 PMC2989900

[B17] Mancinelli A. (1994). “The physiology of phytochrome action,” in Photomorphogenesis in plants. Editors Kendrick R. E. Kronenberg G. M. H. (Dordrecht: Kluwer Academic Publishers.), 211–269.

[B18] Minguet S. Swamy M. Alarcón B. Luescher I. F. Schamel W. W. A. (2007). Full activation of the T cell receptor requires both clustering and conformational changes at CD3. Immunity 26, 43–54. 10.1016/j.immuni.2006.10.019 17188005

[B19] Mukougawa K. Kanamoto H. Kobayashi T. Yokota A. Kohchi T. (2006). Metabolic engineering to produce phytochromes with phytochromobilin, phycocyanobilin, or phycoerythrobilin chromophore in *Escherichia coli* . FEBS Lett. 580, 1333–1338. 10.1016/j.febslet.2006.01.051 16458890

[B20] Rockwell N. C. Su Y.-S. Lagarias J. C. (2006). Phytochrome structure and signaling mechanisms. Annu. Rev. Plant Biol. 57, 837–858. 10.1146/annurev.arplant.56.032604.144208 16669784 PMC2664748

[B21] Sánchez M. F. Tampé R. (2022). Ligand-independent receptor clustering modulates transmembrane signaling: A new paradigm. Trends Biochem. Sci. 48, 156–171. 10.1016/j.tibs.2022.08.002 36115755

[B22] Schamel W. W. A. Alarcon B. Höfer T. Minguet S. (2017). The allostery model of TCR regulation. J. Immunol. 198, 47–52. 10.4049/jimmunol.1601661 27994168

[B23] Schamel W. W. A. Arechaga I. Risueño R. M. van Santen H. M. Cabezas P. Risco C. (2005). Coexistence of multivalent and monovalent TCRs explains high sensitivity and wide range of response. J. Exp. Med. 202, 493–503. 10.1084/jem.20042155 16087711 PMC2212847

[B24] Schamel W. W. Alarcon B. Minguet S. (2019). The TCR is an allosterically regulated macromolecular machinery changing its conformation while working. Immunol. Rev. 291, 8–25. 10.1111/imr.12788 31402501

[B25] Smith R. W. Helwig B. Westphal A. H. Pel E. Hörner M. Beyer H. M. (2016). Unearthing the transition rates between photoreceptor conformers. BMC Syst. Biol. 10, 110. 10.1186/s12918-016-0368-y 27884151 PMC5123409

[B26] Swamy M. Beck-Garcia K. Beck-Garcia E. Hartl F. A. Morath A. Yousefi O. S. (2016). A cholesterol-based allostery model of T cell receptor phosphorylation. Immunity 44, 1091–1101. 10.1016/j.immuni.2016.04.011 27192576

[B27] Taylor M. J. Husain K. Gartner Z. J. Mayor S. Vale R. D. (2017). A DNA-based T cell receptor reveals a role for receptor clustering in ligand discrimination. Cell 169, 108–119.e20. 10.1016/j.cell.2017.03.006 28340336 PMC6934412

[B28] Thomas O. S. Hörner M. Weber W. (2020). A graphical user interface to design high-throughput optogenetic experiments with the optoPlate-96. Nat. Protoc. 15, 2785–2787. 10.1038/s41596-020-0349-x 32709989

[B29] Tischer D. K. Weiner O. D. (2019). Light-based tuning of ligand half-life supports kinetic proofreading model of T cell signaling. Elife 8, e42498. 10.7554/eLife.42498 30947808 PMC6488292

[B30] Toettcher J. E. Weiner O. D. Lim W. A. (2013). Using optogenetics to interrogate the dynamic control of signal transmission by the Ras/Erk module. Cell 155, 1422–1434. 10.1016/j.cell.2013.11.004 24315106 PMC3925772

[B31] von Horsten S. Straß S. Hellwig N. Gruth V. Klasen R. Mielcarek A. (2016). Mapping light-driven conformational changes within the photosensory module of plant phytochrome B. Sci. Rep. 6, 34366. 10.1038/srep34366 27694986 PMC5046071

[B32] Wagner J. R. Brunzelle J. S. Forest K. T. Vierstra R. D. (2005). A light-sensing knot revealed by the structure of the chromophore-binding domain of phytochrome. Nature 438, 325–331. 10.1038/nature04118 16292304

[B33] Yousefi O. S. Günther M. Hörner M. Chalupsky J. Wess M. Brandl S. M. (2019). Optogenetic control shows that kinetic proofreading regulates the activity of the T cell receptor. Elife 8, e42475. 10.7554/eLife.42475 30947807 PMC6488296

[B34] Yousefi O. S. Hörner M. Wess M. Idstein V. Weber W. Schamel W. (2020). Optogenetic tuning of ligand binding to the human T cell receptor using the opto-ligand-TCR system. Bio Protoc. 10, e3540. 10.21769/bioprotoc.3540 PMC7842703 33659514

[B35] Yousefi O. S. Ruggieri M. Idstein V. von Prillwitz K. U. Herr L. A. Chalupsky J. (2021). Cross-TCR antagonism revealed by optogenetically tuning the half-life of the TCR ligand binding. Int. J. Mol. Sci. 22, 4920. 10.3390/ijms22094920 34066527 PMC8124730

[B36] Zakeri B. Fierer J. O. Celik E. Chittock E. C. Schwarz-Linek U. Moy V. T. (2012). Peptide tag forming a rapid covalent bond to a protein, through engineering a bacterial adhesin. Proc. Natl. Acad. Sci. U. S. A. 109, E690–E697. 10.1073/pnas.1115485109 22366317 PMC3311370

