# Supplementary material for: Opto-APC: Engineering of cells that display phytochrome B on their surface for optogenetic studies of cell-cell interactions
Source: Front Mol Biosci. 2023 Feb 20;10:1143274. doi: 10.3389/fmolb.2023.1143274 (PMC10016228; doi:10.3389/fmolb.2023.1143274)
Supplement: Supplementary file 1 [file DataSheet1.PDF]

Supplementary Material

Russ et al.,

Engineering of cells that displays Phytochrome B on their surface for optogenetic studies of cell-cell interactions

1 Supplementary Figures

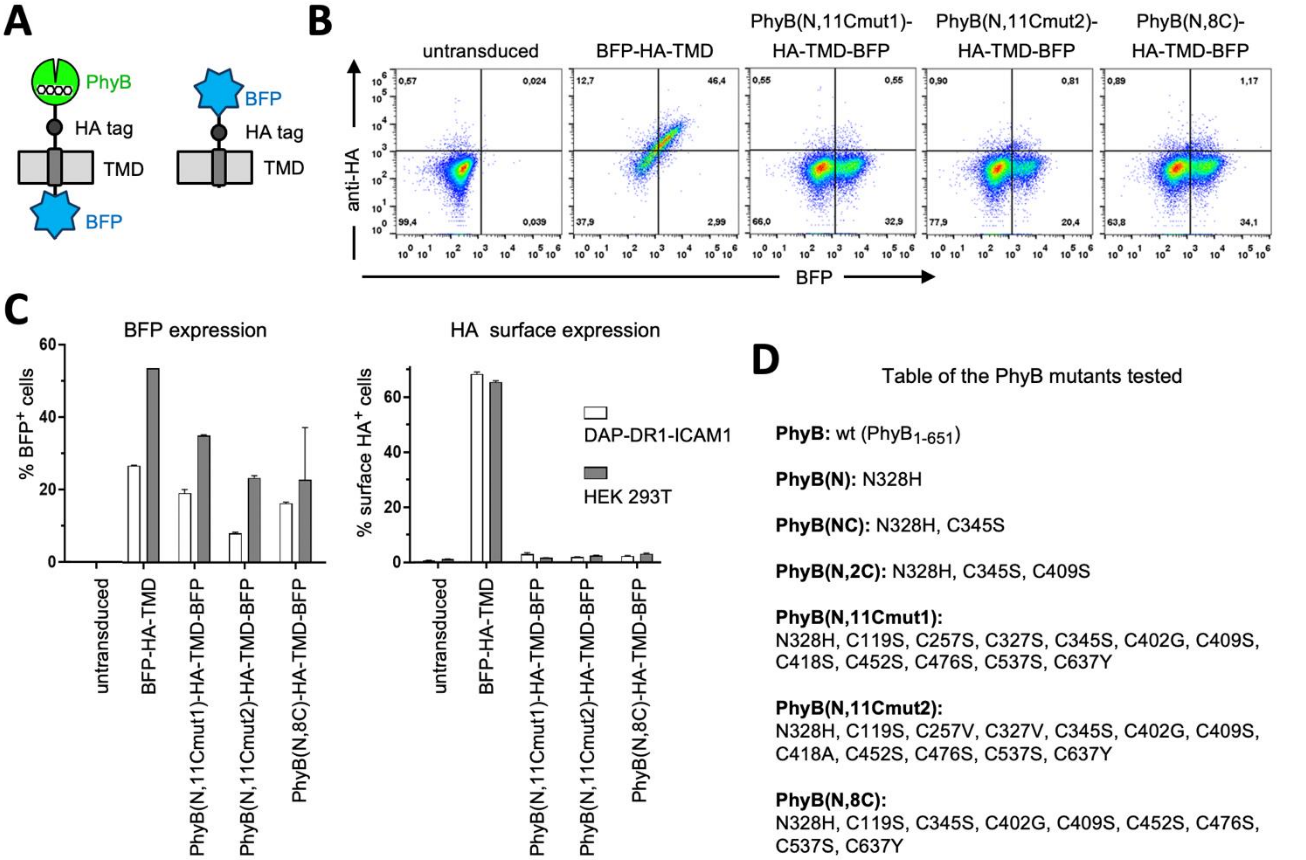

Suppl. Figure 1. PhyB cannot be expressed as a transmembrane protein on the cell surface
(A) Schematic picture of PhyB-HA-TMD-BFP and the control BFP-HA-TMD construct (the signal peptides are not shown). **(B)** HEK 293T cells were lentivirally transduced and the cells incubated overnight with 15 µM PCB to allow formation of a functional PhyB. Cells were stained with an AlexaFluor647-coupled anti-HA tag antibody measured by flow cytometry. The fluorescence of AlexaFluor647and BFP is shown. Although the proteins were expressed as seen by the BFP fluorescence, we could not detect localisation on the cell surface as the cells were HA-negative (except for the BFP-HA-TMD control). **(C)** Quantification of (B) and also including DAP-DR1-ICAM1 cells in addition to HEK 293T. **(D)** The PhyB mutants tested are shown. In one construct only the two outward facing Cys (C345 and C409) in addition to the glycosylation sequence (N-X-S/T) harbouring Asp (N328) were mutated. C357 binds to PCB, therefore, this Cys was never mutated.
We also failed to bind GFP-PIF to the membrane-bound PhyB-transduced cells and failed to stimulate GFP-PIF-TCR expressing Jurkat cells, showing again that functional PhyB was not expressed on the cell surface. In some PhyB constructs we also omitted the HA tag and placed YFP to the ectodomain instead of BFP to the cytoplasmic side. We also tested the construct from (Yüz et al., 2018). Unfortunately, all these constructs were also not expressed on the cell surface.

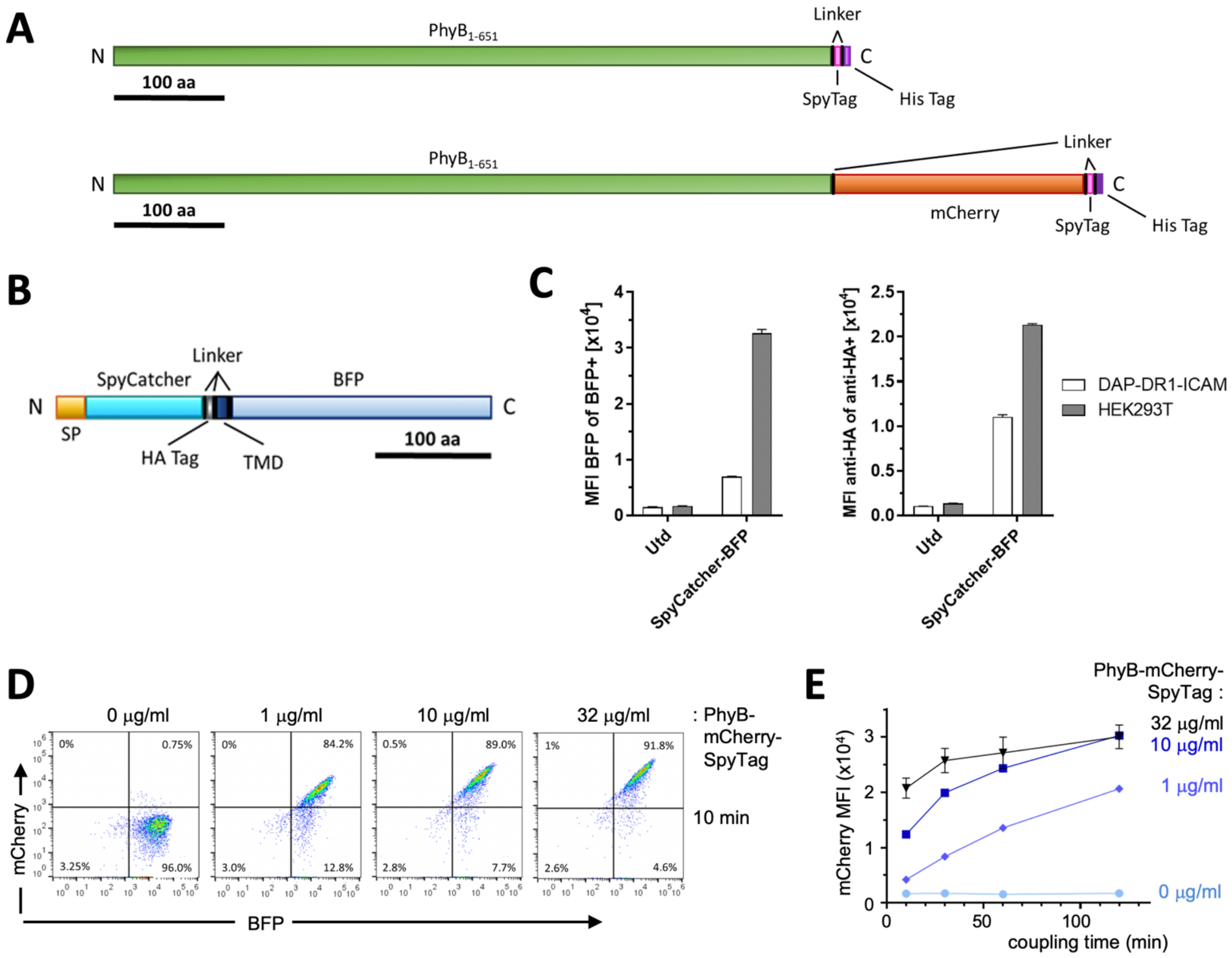

Suppl. Figure 2. Engineering of the PhyB$_{1-651}$-SpyTag and SpyCatcher-TMD-BFP system.
(A) Schematic depiction of the cDNAs encoding for the PhyB$_{1-651}$-SpyTag constructs without and with mCherry. **(B)** Schematic depiction of the cDNA encoding for the SpyCatcher-TMD-BFP construct (SP: signal peptide, TMD: transmembrane domain, BFP: blue fluorescent protein). **(C)** Bar graphs of the mean fluorescence intensities (MFI) of BFP and the anti-HA tag stain in DAP-DR1-ICAM1 and HEK 293T cells expressing SpyCatcher-TMD-BFP or not (untransduced). **(D)** SpyCatcher-TMD-BFP expressing HEK 293T cells were incubated for 10 min with 0, 1, 10 and 32 µg/ml of PhyB-mCherry-SpyTag at 37°C. After washing cells were analysed by flow cytometry. Dot plots displaying the fluorescence intensities of mCherry and BFP are shown. **(E)** The experiment of (D) was done using different incubation times (10, 30, 60 and 120 min), and the mCherry fluorescence intensity, indicative of PhyB-mCherry-SpyTag binding to the cells is shown.

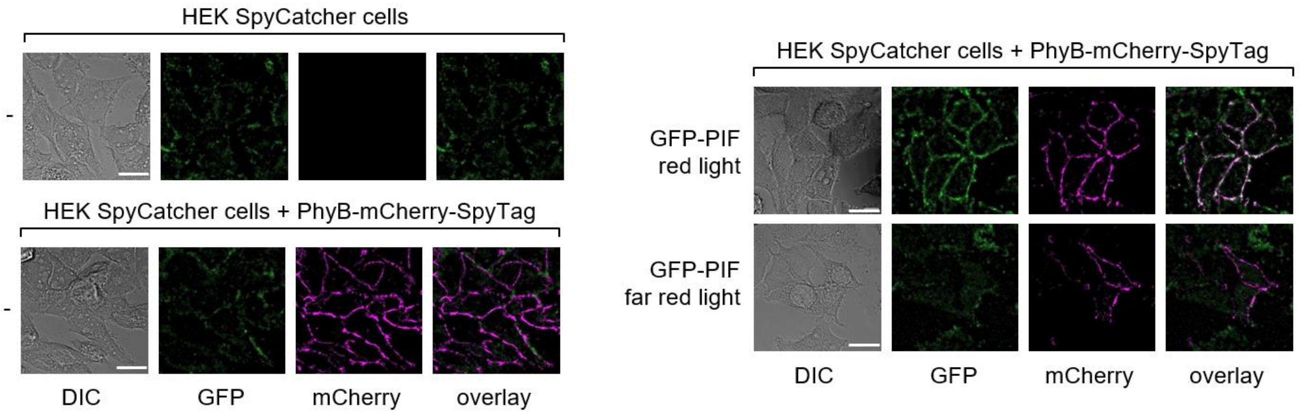

Suppl Figure 3. Binding of GFP-PIF to adherent SpyCatcher-TMD-BFP-expressing HEK-293T cells

SpyCatcher-TMD-BFP-expressing HEK-293T cells loaded or not with 10 µg/ml PhyB-mCherry-SpyTag for 30 min at 37°C and 5% CO_2, as indicated. Then 100 nM GFP-PIF was added (right panels) or not (left panels), and cells were illuminated with red, 660 nm or with far red, 740 nm light and incubated for 20 min in the dark prior to fixation. Cells were recorded by confocal microscopy. Scale bars represent 20 µm (n>3).

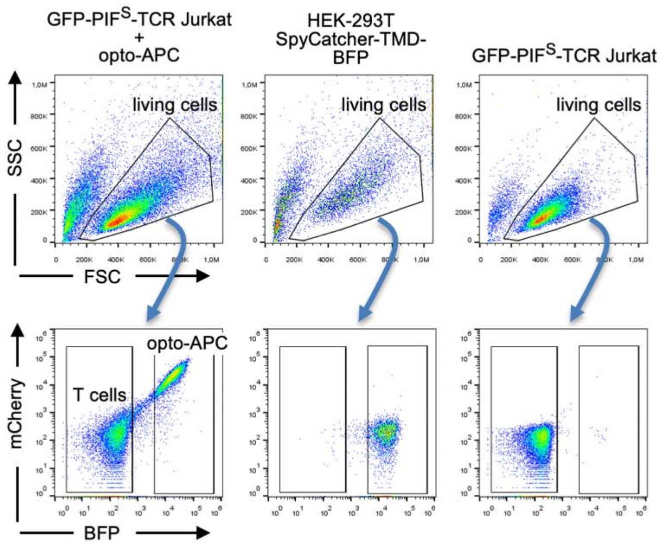

Suppl. Figure 4. Gating strategy to identify the GFP-PIF^S-TCR-expressing Jurakt T cells

The cells given were measured by flow cytometry. We first gated in the forward scatter (FSC) and side scatter (SSC) channels on the living cells (upper row). The living cells were then displayed with the mCherry fluorescence intensity on the Y-axis and the BFP fluorescence intensity on the X-axis (lower panel). The T cells are the mCherry and BFP negative cells and can thus be distinguished from the opto-APCs. The latter are BFP-positive, due to their expression of SpyCatcher-TMD-BFP, and mCherry-positive, due to their coupling to PhyB-mCherry-SpyTag.

2 Cloning of the constructs used in this study

The plasmids in Table 1were created using the standard molecular cloning PCR, enzymatic digestion, and ligation or Gibson assembly reaction. Table 2 shows the cloning strategies of the new plasmids. All used oligonucleotides are listed in Table 3. As backbone for plasmids used for protein expression in *E. coli* pCDF (Novagen) was used. The backbone for pMH1411 was pRSET (Thermofisher). For the lentiviral plasmids pCDH (System Bioscience) was used as a backbone. All plasmids were verified by restriction enzyme digestion and Sanger sequencing.

Table 1: List of the newly generated plasmids

Plasmid	Expressed protein	Description
pOSY094	PhyB-YFP	HA-PhyB$_{1-908}$-YFP-myc-TMD
pOSY095	PhyB	PhyB$_{1-651}$-TMD (MHCI)
pOSY096	PhyB-BFP	PhyB$_{1-651}$-TMD(MHCI)-moxBFP
pOSY097	PhyB(N)	PhyB$_{1-651}$N328H-TMD(MHCI)
pOSY098	PhyB(NC)	PhyB$_{1-651}$N328H_C345S-TMD(MHCI)
pOSY099	PhyB(NCx2)	PhyB(1-651)N328H_C345S_C409S-TMD(MHCI)
pOSY100	PhyB(NC)-BFP	PhyB$_{1-651}$N328H-TMD(MHCI)-moxBFP
pOSY101	PhyB(N2xC)-BFP	PhyB$_{1-651}$N328H_C345S_C409S-TMD(MHCI)-moxBFP
pOSY110	BFP	moxBFP fused to HA tag and MHCI transmembrane domain
pOSY111	PhyB(N11xC-mut1)-BFP	PhyB$_{1-651}$ with mutations N328H, C119S, C257S, C327S, C345S, C402G, C409S, C418S, C452S, C476S, C537S, C637Y, HA tag, TMD (MHCI), and moxBFP
pOSY112	PhyB(N11xC mut2)-BFP	PhyB$_{1-651}$ with mutations N328H, C119S, C257V, C327V, C345S, C402G, C409S, C418A, C452S, C476S, C537S, C637Y, HA tag, TMD (MHCI), and moxBFP
pOSY113	PhyB(N8xC)-BFP	PhyB$_{1-651}$ with mutations N328H, C119S, C345S, C402G, C409S, C452S, C476S, C537S, C637Y, HA tag, TMD (MHCI), and moxBFP
pOSY114	SpyTag-BFP	SpyTag003 fused to HA tag, TMD (MHCI), and moxBFP
pOSY115	SpyCatcher-BFP	SpyCatcher003 fused to HA tag, TMD (MHCI), and moxBFP
pOSY116	PhyB-SpyTag	PhyB$_{1-651}$ with mCherry, SpyTag003, His(6), Heme oxygenase, and pcyA
pOSY117	PhyB-mCherry-SpyTag	PhyB$_{1-651}$ with SpyTag003, His(6), Heme oxygenase, and pcyA
pMH1411	moxGFP-Linker-PIF6(1-100, C9S, C10S)-His6	

The sequences of pOSY115 and pOSY117 can be found in Hörner et al. 2021 (Hörner et al., 2021).

Table 2: Cloning strategies of the newly generated plasmids

Plasmid	Cloning strategy
pOSY094	Gibson of two fragments: 1) pDisplay-PhyB (SY_16) EcoRI-NotI fragment, 2) pOSY082 NotI-EcoRI fragment

Table 2: Cloning strategies of the newly generated plasmids

Plasmid	Cloning strategy
pOSY095	Gibson of three fragments: 1) pOSY082 BamHI-EcoRI fragment, 2) PCR with O233 fw and O234 rv from 3) PCR with O231 fw and O232 rv from pMH17
pOSY096	Gibson of four fragments: 1) pOSY082 BamHI-EcoRI fragment, 2) PCR with O233 fw and O235 rv from 3) PCR with O231 fw and O232 rv from pMH17, 3) PCR with O236 fw and O237 rv from moxBFP
pOSY097	Gibson of three fragments: 1) pOSY095 BamHI digest, 2) PCR with O238 fw and O239 rv from pOSY095, 3) PCR with O240 fw and O241 rv from pOSY095
pOSY098	Gibson of three fragments: 1) pOSY095 BamHI digest, 2) PCR with O238 fw and O242 rv from pOSY097, 3) PCR with O243 fw and O241 rv from pOSY095
pOSY099	Gibson of three fragments: 1) pOSY095 BamHI digest, 2) PCR with O238 fw and O244 rv of pOSY098, 3) PCR with O245 fw and O241 rv of pOSY095
pOSY100	Gibson of two fragments: 1) pOSY097 EcoRI-BclI fragment, 2) pOSY096 BclI-EcoRI fragment
pOSY101	Gibson of two fragments: 1) pOSY099 EcoRI-BclI fragment, 2) pOSY096 BclI-EcoRI fragment
pOSY110	Gibson of three fragments: 1) pOSY095 BamHI-ApaI fragment, 2) PCR with O268 fw and O241 rv from pOSY095, 3) PCR with O266 fw and O267 from pOSY101
pOSY111	Gibson of two fragments: 1) SP PhyB TMD moxBFP gBlock, 2) pOSY101 AdeI-ApaI fragment
pOSY112	Gibson of three fragments: 1) pOSY111 PstI-EcoRV fragment, 2) PCR with O270 fw and O271 rv from pOSY111, 3) PCR with O272 fw and O273 rv from pOSY111
pOSY113	Gibson of three fragments: 1) pOSY111 PstI-EcoRV fragment, 2) PCR with O274 fw and O271 rv from pOSY111, 3) PCR with O275 fw and O276 rv from pOSY111
pOSY114	Gibson of three fragments: 1) pOSY101 AdeI-ApaI fragment, 2) PCR with O279 fw and O278 rv from 3) PCR with O277 fw and O278 rv from SP PhyB TMD moxBFP gBlock
pOSY115	Gibson of three fragments: 1) pOSY101 AdeI-ApaI fragment, 2) PCR with O281 fw and O282 rv from SpyCatcher gBlock ,3) PCR with O280 fw and O278 rv from SP PhyB TMD moxBFP gBlock
pOSY116	Gibson of three fragments: 1) pMH1105 NotI-EcoRV fragment, 2) PCR with O246 fw and O284 rv from 3) PCR with O246 fw and O283 rv from pOSY107
pOSY117	Gibson of three fragments: 1) pMH1105 NotI-EcoRV fragment, 2) PCR with O246 fw and O284 rv from 3) PCR with O246 fw and O285 rv from pOSY107
pMH1411	Gibson of two fragments: 1) PCR with oMH1432 and oMH220 from pMH613, 2) PCR with oMH1430 and oMH1431 from pOSY076

Table 3: List of oligonucleotides

Name	Sequence (5' to 3')
O019 rv	CAGCGTATCCACATAGCG
O209 fw	TATAAGTGCAGTAGTCGCC
O231 fw	ATGGCCGTCATGGCGCCCCGAACCCTCCTCCTGCTACTCTCGGGGGCCCTG GCCCTGACCCAGACCTGGGCG ATGGTTTCCGGAGTCGGG

Table 3: List of oligonucleotides

Name	Sequence (5' to 3')
O232 rv	CAAGGAGAACCAGGCCAGCAATGATGCCCACGATGGGGATGGTGGGCTGG GAAGACAGTCCAGAACCGCCTCCAGAACCGCCTGCACCTAACTCATCAAC CCC
O233 fw	CGCCAGAACACAGTCTAGAGCTAGCGAATTCGAAGCCACCATGGCCGTCA TGGCGC
O234 rv	CAGAGGTTGATTGTCGACGCGGCCGCGGATCCTCATGAGCTCTTCCTCCTC CACATCACGGCAGCGACCACAGCTCCAGTGATCACAGCTCCAAGGAGAAC CAGGCCAGC
O235 rv	AGAACCGCCTGAGCTCTTCCTCCTCCACATCACGGCAGCGACCACAGCTCC AGTGATCACAGCTCCAAGGAGAACCAGGCCAGC
O236 fw	ATGTGGAGGAGGAAGAGCTCAGGCGGTTCTGGAGGCGGTTCTGGAATGGT GAGCAAGGGCGAG
O237 rv	ATCCAGAGGTTGATTGTCGACGCGGCCGCGGATCCTCACTTGTACAGCTCG TCCATGCC
O238 fw	TCGAGAGATTACCTTGTTAAATCCG
O239 rv	AACAGGTGTGGCATGGCAATCTACTATCATTCGGACAC
O240 fw	ATGATAGTAGATTGCCATGCCACACCTGTTCTTG
O241 rv	TCACAAATTTTGTAATCCAGAGGTTG
O242 rv	GTAGAACCAACCAAGG ACATAGACTGAGTTAGCCTATCG
O243 fw	TAACTCAGTCTATGTCCTTGGTTGGTTCTACTCTTAGGG
O244 rv	AGCGGAAACGGTATGGAGCGAGAAGAAGTGTGATGG
O245 fw	ACACTTCTTCTCGCTCCATACCGTTTCCGCTAAGG
O246 fw	AGCCAGCTGCAGGCAC
O248 fw	AGCATGGAGAAGTTGTAGC
O249 fw	GTTCAGAGCCAGAAACTG
O253 fw	TGTTGTTCAGCCGTGTC
O266 fw	GCCCCGAACCCTCCTCCTGCTACTCTCGGGGGCCCTGGCCCTGACCCAGAC CTGGGCGGTGAGCAAGGGCGAGGAG
O267 rv	CTTGTACAGCTCGTCCATGCC
O268 fw	ATCACTCTCGGCATGGACGAGCTGTACAAGGGAGGCAGCGGTTACCCATA CGATGTTCCAGATTACGCT GGCGGTTCTGGACTGTC
O269 fw	CCAGAAATTGGCCGTTAG
O270 fw	TCTCAGCTTCAGGCTCTTCCTGGTGGAGATATCAAGCTTTTGGTTGACACT GTCGTGGAAAGTGTG
O271 rv	ATCTACTATCATCCGGACTCTGTTCTGC
O272 fw	GCAGAACAGAGTCCGGATGATAGTAGATGTCCATGCCACACCTGTTCTTG
O273 rv	TGCAAAGCCAGCTGCAATTCCATATTCAACTGCAGACCGAAAGCCTGCATC AAAAACTC AGCAGCATATCTCAACGGAAACGGTATG
O274 fw	TCTCAGCTTCAGGCTCTTCCTGGTGGAGATATCAAGCTTTTG TGTGACACTGTCGTGGAAAGTGTG
O275 fw	GCAGAACAGAGTCCGGATGATAGTAGAT TGCCATGCCACACCTGTTCTTG

Table 3: List of oligonucleotides

Name	Sequence (5' to 3')
O276 rv	TGCAAAGCCAGCTGCAATTCCATATTCAACTGCAGACCGAAAGCCTGCATC AAAAACTC ACAAGCATATCTCAACGGAAACGGTATG
O277 fw	GGAGGAAGCGGCCGTGGCGTGCCTCATATCGTGATGGTGGACGCCTACAA GCGTTACAAGGGCGGTTCTGGATACCCATAC
O278 rv	GTGGTCGGCGAGCTG
O279 fw	GCGCCCCGAACCCTCCTCCTGCTACTCTCGGGGGCCCTGGCCCTGACCCAG ACCTGGGCGGGAGGAAGCGGCCG
O280 fw	GGCGATGCACACACC GGCGGTTCTGGATACCCATAC
O281 fw	GCGCCCCGAACCCTCCTCCTGCTACTCTCGGGGGCCCTGGCCCTGACCCAG ACCTGGGCGGGAGGAAGCGGC GTGACCACACTGTCCGGAC
O282 rv	GTATCCAGAACCGCC GGTGTGTGCATCGCCTTC
O283 rv	TCCAGAACCGCCCTTGTAACGCTTGTAGGCGTCCACCATCACGATATGAGG CACGCCACGGCTACCAGAACCTGCACCC
O284 rv	CTTTCTGTTCGACTTAAGCATTATGCGGCCGCTTAATGGTGATGGTGATGA TGTCCAGAACCGCCCTTGTAACG
O285 rv	TCCAGAACCGCCCTTGTAACGCTTGTAGGCGTCCACCATCACGATATGAGG CACGCCACGACCTGATCCACCCTTGTACAGC
O286 rv	GGACAGTGTGGTCAC GCTACCAGAACCTGCACCC
O287 fw	GCAGGTTCTGGTAGC GTGACCACACTGTCCGGAC
O288 rv	CTTTCTGTTCGACTTAAGCATTATGCGGCCGCTTAATGGTGATGGTGATGA TGTCCAGAACCGCCGGTGTGTGCATCGCCTTC
O289 rv	GGACAGTGTGGTCAC ACCTGATCCACCCTTGTACAGC
O290 fw	AAGGGTGGATCAGGT GTGACCACACTGTCCGGAC
oMH220 rv	CATATGTATATCTCCTTCTTAAAGTTAAAC
oMH1430 fw	AATAATTTTGTTTAACTTTAAGAAGGAGATATACATATGGTGTCCAAGGGC GAGGAGC
oMH1431 rv	CTTGTACAGCTCGTCCATGCCG
oMH1432 fw	CGGCATGGACGAGCTGTACAAG

References

Hörner, M., Jerez-Longres, C., Hudek, A., Hook, S., Yousefi, O. S., Schamel, W. W. A., et al. (2021). Spatiotemporally confined red light-controlled gene delivery at single-cell resolution using adeno-associated viral vectors. *Sci Adv* 7. doi: 10.1126/sciadv.abf0797.

Yüz, S. G., Ricken, J., and Wegner, S. v (2018). Independent Control over Multiple Cell Types in Space and Time Using Orthogonal Blue and Red Light Switchable Cell Interactions. *Adv Sci (Weinh)* 5, 1800446. doi: 10.1002/advs.201800446.
